# Characteristics of Patients with Smear-Negative Pulmonary Tuberculosis (TB) in a Region with High TB and HIV Prevalence

**DOI:** 10.1371/journal.pone.0147933

**Published:** 2016-01-25

**Authors:** Leandro Cruz Campos, Marcos Vinícius Vieira Rocha, Denise Maria Cunha Willers, Denise Rossato Silva

**Affiliations:** 1 Programa de Pós-Graduação em Ciências Pneumológicas, UFRGS, Porto Alegre, Brazil; 2 Faculdade de Medicina, Universidade Federal do Rio Grande do Sul, Porto Alegre, Brazil; 3 Hospital de Clínicas de Porto Alegre, Porto Alegre, Brazil; UCL Institute of Child Health, University College London, UNITED KINGDOM

## Abstract

**Introduction:**

Smear-negative pulmonary TB (SNPT) represents 30–60% of all pulmonary TB cases. The mortality of these patients can reach 25% in populations with high prevalence of HIV infection, and 10–20% of TB transmission at the population level are attributable to SNPT cases.

**Methods:**

We conducted a retrospective study to evaluate epidemiological, clinical, and radiological characteristics of patients with SNPT and to compare these with patients who were diagnosed as having smear-positive pulmonary TB (SPPT). All adult patients (≥ 18 years old) with a positive culture for *Mycobacterium tuberculosis*, and a diagnosis of pulmonary TB were included in the study.

**Results:**

198 patients met the inclusion criteria (positive culture for *Mycobacterium tuberculosis*) and were included in the analysis. Of these patients, 69 (34.8%) were smear positive (SPPT) and 129 (65.2%) were smear negative (SNPT). In univariate analysis, cough, dyspnea, and hemoptysis were less frequent in SNPT patients in comparison with SPPT patients. In a multivariate model, having no cough and no radiographic pattern typical of TB were the characteristics independently associated with a diagnosis of SNPT.

**Conclusions:**

We found a very high prevalence of SNPT among patients with TB in a setting with high TB and HIV prevalence. The absence of cough in the presence of other symptoms suggestive of TB, and having no radiographic pattern typical of TB where independent predictors of SNPT.

## Introduction

Pulmonary tuberculosis (TB) is still one of the most common infectious diseases in the world. It is the second leading cause of death from an infectious disease worldwide, after the human immunodeficiency virus (HIV) [1]. Brazil is ranked 16^th^ among the 22 high-burden countries that collectively account for 80% of TB cases globally, with an incidence of 33.5 cases/100,000 inhabitants/year in 2014. The city of Porto Alegre has the highest incidence of TB in the country (99.3 cases/100,000 inhabitants/year in 2014), and 28.0% of TB patients are coinfected with HIV [2].

For the diagnosis of TB, the World Health Organization (WHO) recommends initial smear microscopy for the detection of acid-fast bacilli. However, this is not a sensitive technique, and only 57% of new cases of pulmonary TB reported were smear-positive [1]. Sputum culture for mycobacteria has higher diagnostic yield in comparison with smear microscopy. Nevertheless, culture is more time-consuming in its results—the turn-around time is about 2–8 weeks—so it is less useful to guide the clinical decision-making process [3]. Thus the treatment is often carried out empirically, based on clinical criteria, and can result in unnecessary costs and toxicities.

Smear-negative pulmonary TB (SNPT) represents 30–60% of all pulmonary TB cases, according to region [4]. In Brazil, 24–30% of cases of pulmonary TB in adults are SNPT [5]. The mortality of these patients can reach 25% in populations with high prevalence of HIV infection, which may be largely related to delay in diagnosis [6,7]. Furthermore, although smear-positive patients are considered to be more infectious, 10–20% of TB transmission at the population level are attributable to SNPT cases [8–10].

As much as possible, patients should be correctly diagnosed for the early initiation of treatment for SNPT. On the other hand, the treatment of those without the disease should be avoided. In the absence of rapid, simple, and accurate TB diagnostic tools for SNPT, frequently occurring clinical features are significant in supporting the diagnosis of SNPT, especially in areas with high TB and HIV infection. The aim of this study was therefore to identify the prevalence and the epidemiological characteristics, clinical presentation, and radiological findings of patients with SNPT and to compare these with patients who were diagnosed as having smear-positive pulmonary TB (SPPT).

## Methods

### Study Design and Location

We conducted a retrospective study to evaluate epidemiological, clinical, and radiological characteristics of patients with SNPT and to compare these with patients diagnosed with SPPT. The study location was Hospital de Clínicas de Porto Alegre (HCPA), a general, tertiary care, university-affiliated hospital with 750 beds and approximately 29,000 hospitalizations/year. It is a reference center for HIV in south of Brazil. The ethics committee at Hospital de Clínicas de Porto Alegre has approved access to patient records on January 22^nd^, 2013 (number 13–0024). Patient confidentiality has been maintained.

### Patients and Data Collection

The records of all adult patients (≥ 18 years old) with a positive culture for *Mycobacterium tuberculosis* during the study period (January 2013—June 2015) were analyzed. We included only patients with a diagnosis of pulmonary TB, according to the Brazilian Guidelines for Tuberculosis [3], and a positive culture in spontaneous sputum, induced sputum, or bronchoalveolar lavage. Patients with extrapulmonary TB were excluded from the study. Patients with a positive culture and a negative smear microscopy were classified as SNPT, and patients with a positive culture and a positive smear microscopy were classified as SPPT [4].

The following data were collected from patient records using a standardized data extraction tool: demographic data (sex, age, ethnicity, years of schooling), behavioral data (smoking status, alcohol abuse, injection drug use), and medical history (clinical form of TB, symptoms at admission, diagnostic methods, presence of comorbidities, prior TB treatment, drug regimen, interval from hospital admission until diagnosis, length of hospital stay, intensive care unit [ICU] admission, duration of mechanical ventilation, and hospitalization outcome [death or discharge]). A current smoker was defined as reporting smoking at least 100 cigarettes in their lifetime, and at the time of the survey were smoking at least one day a week. A former smoker was defined as reporting smoking at least 100 cigarettes in their lifetime but who, at the time of the survey, did not smoke at all. Never smoked reported having smoked < 100 cigarettes in their lifetime [11]. Alcohol abuse was defined as daily consumption of at least 30 grams (equivalent to a pint and a half of 4% beer) for men and 24 grams (equivalent to a 175 ml glass of wine) for women. An independent physician analyzed the chest X-rays (CXRs) and classified them as typical of TB (the presence of nodular, alveolar, or interstitial infiltrates predominantly affecting the zones above the clavicles or upper zones; the presence of cavitation affecting the upper zones or the apical segment of the lower lobe) or compatible with TB (enlarged hilar nodes, pneumonic lesion, atelectasis, mass lesion, miliary), according to previously described guidelines [12].

### Statistical Analysis

Data analysis was performed using SPSS 18.0 (Statistical Package for the Social Sciences, Chicago, Illinois). Data were presented as number of cases, mean ± standard deviation (SD), or median with interquartile range (IQR). Categorical comparisons were performed by chi-square test using Yates’s correction if indicated or by Fisher’s exact test. Continuous variables were compared using the *t*-test or Wilcoxon test. Multivariate logistic regression analysis was used to evaluate factors associated with a diagnosis of SNPT, using selection of factors associated (p ≤ 0.20) with this diagnosis in univariate analysis or those known to have clinical significance. Stepwise regression models were also examined to evaluate the possible collinearity among the predictors. The predictors selected in the final model were based on both statistical and clinical significance. The goodness of fit of the multiple logistic regression models was assessed using the Hosmer-Lemeshow test. Odds ratios (ORs) and nominal 95% confidence intervals (CIs) were presented. A two-sided p value < 0.05 was considered significant for all analyses. Sample size calculation was based on the prevalence of SNPT in Brazil (24–30%) [5]. Considering the amplitude of the confidence interval of 0.15 and a 95% confidence level, we estimated a sample size of 128 patients.

## Results

During the study period, 198 patients met the inclusion criteria [positive culture for *Mycobacterium tuberculosis* in spontaneous sputum (n = 49), induced sputum (n = 54), or bronchoalveolar lavage (n = 95)] and were included in the analysis. Out of these patients, 69 (34.8%) were smear positive (SPPT) and 129 (65.2%) were smear negative (SNPT). The characteristics of the study population are shown in Table 1.

**Table 1:** Characteristics of patients with SNPT and SPPT.

Characteristics	SNPT (n = 129)	SPPT (n = 69)	p value
**Demographic characteristic**			
Age* (yr), mean ± SD	46.6 ± 17.1	46.0 ± 16.9	0.833
Male sex*, n (%)	74 (57.4)	47 (68.1)	0.185
White ethnicity, n (%)	98 (76.0)	57 (82.6)	0.369
< 8 years of schooling, n (%)	86 (66.7)	42 (60.9)	0.511
**Current smokers,** n (%)	47 (36.4)	24 (34.8)	0.940
Former smokers*, n (%)	19 (14.7)	16 (23.2)	0.171
**Never smokers,** n (%)	63 (48.8)	29 (42.0)	0.374
**Alcohol abuse,** n (%)	33 (25.6)	19 (27.5)	0.898
**Drug use,** n (%)	32 (24.8)	17 (24.6)	0.999
**Symptoms**			
Cough*, n (%)	71 (55.0)	54 (78.3)	0.002
Night sweats, n (%)	39 (30.2)	19 (27.5)	0.815
Fever, n (%)	59 (45.7)	37 (53.6)	0.363
Weight loss*, n (%)	69 (53.5)	45 (65.2)	0.150
Dyspnea*, n (%)	35 (27.1)	32 (46.4)	0.010
Hemoptysis*, n (%)	4 (3.1)	9 (13.0)	0.013
**Duration of symptoms before admission (days)**^§^,	30 (18.8–97.5)	30 (20–90)	0.724
median (interquartile range)			
**Previous TB,** n (%)	20 (15.5)	16 (23.2)	0.253
**Comorbidities**			
HIV positive, n (%)	53 (41.1)	25 (36.2)	0.608
Diabetes mellitus, n (%)	13 (10.1)	5 (7.2)	0.688
**Radiographic patterns**			
Typical of TB*, n (%)	75 (58.1)	50 (72.5)	0.066
Compatible with TB*, n (%)	54 (41.9)	19 (27.5)	0.083
**Inpatient,** n (%)	91 (70.5)	56 (81.2)	0.145
ICU stay**, n (%)	13 (15.3)	13 (23.2)	0.335
Mechanical ventilation**, n (%)	14 (16.3)	13 (23.2)	0.418
In-hospital mortality**, n (%)	7 (6.7)	9 (16.4)	0.096
Length of hospital stay (days)**, median (interquartile range)	20 (10–49)	14.5 (7–26)	0.010

SNPT: smear negative pulmonary tuberculosis; SPPT: smear positive pulmonary tuberculosis; TB: tuberculosis; HIV: human immunodeficiency virus; ICU: intensive care unit.

* Variables included in multivariate analysis.

** Data available only for inpatients (n = 91 for SNPT group and n = 56 for SPPT group).

^§^ Data available for 151 patients.

The study subjects had a mean age of 46.6 ± 17.1 years in SNPT group and 46.0 ± 16.9 years in SPPT group (p = 0.833). There were no statistically significant differences between study groups according to gender, ethnicity, smoking, alcohol abuse, and drug use. Among SNPT there were 53 (41.1%) HIV positive patients, and among SPPT there were 25 (36.2%) HIV positive patients (p = 0.608).

Cough was the most common symptom in both groups, although it was more frequent in SPPT (54 [78.3%]) than in SNPT (71 [55.0%]) (p = 0.002). Dyspnea and hemoptysis were also more frequent in patients with SPPT (32 [46.4%] and 9 [13.0%], respectively) than in patients with SNPT (35 [27.1%] and 4 [3.1%], respectively) (p = 0.010 and p = 0.013, respectively). The radiographic patterns typical of TB were the most common in both study groups. Patients with SNPT showed a pattern typical of TB less frequently (75 [58.1%]) in comparison with patients with SPPT (50 [72.5%]). However, this difference was not statistically significant in univariate analysis (p = 0.066). As expected, the median length of hospital stay was higher in SNPT patients (20 days [10–49 days]) than in SPPT patients (14.5 days [7–26 days]) (p = 0.010).

Logistic regression analysis estimating the ORs of factors associated with a diagnosis of SNPT was conducted. Age, male sex, former smoker, cough, weight loss, dyspnea, hemoptysis, radiographic pattern typical of TB and compatible with TB were the variables included in the multivariate model. The Hosmer-Lemeshow test showed that the multivariate model demonstrated a good fit (p = 0.684), and there was no evidence for collinearity among the independent variables. The following variables remained significant in the final multivariate model: no coughing (OR 3.10, 95%CI 1.55–6.19, p = 0.001) and no radiographic pattern typical of TB (OR 1.95, 95%CI 1.02–3.76, p = 0.045) (Table 2).

**Table 2:** Multivariate logistic regression model of risk factors associated with SNPT.

Variable	Unadjusted	95% CI	p Value	Adjusted	95% CI	p Value
	OR			OR		
Age, years	0.998	0.981–1.016	0.833	0.994	0.976–1.012	0.507
No coughing	2.068	1.271–3.365	0.002	3.10	1.55–6.19	0.001
No radiographic pattern typical of TB	1.520	0.986–2.345	0.066	1.95	1.02–3.76	0.045

## Discussion

In this retrospective study, we demonstrated a high prevalence of SNPT (65.2%) among patients with TB in a tertiary care hospital in a region with high TB and HIV prevalence. In univariate analysis, cough, dyspnea, and hemoptysis were less frequent in SNPT patients in comparison with SPPT patients. In addition, the median length of hospital stay was higher in SNPT patients than in SPPT patients. In a multivariate model, having no cough and no radiographic pattern typical of TB were the characteristics associated with a diagnosis of SNPT.

SNPT is a relevant clinical and public health problem [6,13,14], especially in high HIV prevalence countries, where the number of patients with SNPT is increasing rapidly [15]. We found a very high prevalence of SNPT in our study. Previous investigations showed that the proportion of cases of SNPT ranged from 10% to 61% of all pulmonary TB cases [8,16–19]. Many referral hospitals are confronted with relatively more SNPT cases than the lower level health facilities [20], and this is an important problem in daily medical practice in hospitals. Treating a patient inappropriately may cause side effects such as drug-induced hepatitis [21]. On the other hand, prompt initiation of treatment is vital considering that SNPT patients have worse outcomes and excessive early mortality compared with SPPT patients [22–26]. Therefore, in order to avoid harmful delays in the establishment of a diagnosis, most cases of SNPT are diagnosed on the basis of the clinical presentation and radiological findings [21].

Regarding signs and symptoms, cough, dyspnea, and hemoptysis were less frequent in SNPT patients in comparison with SPPT patients in our study in univariate analysis. In a multivariate analysis, having no cough remained independently associated with a diagnosis of SNPT. SNPT patients are especially likely to show no or mild respiratory symptoms and systemic manifestations [27]. In a retrospective study [28] based in a chart review of 146 HIV-infected patients, SNPT patients were significantly less likely to present with dyspnea, similarly to our study. Hemoptysis is also less common in these patients and some studies have reported a decreased proportion of patients with cough among SNPT patients [29,30] as we have demonstrated. In a study developed to validate a clinical-radiological score to assess the probability of pulmonary TB in patients suspected of SNPT, productive cough was less frequent in SNPT patients [31]. In another study conducted in Brazil, 551 patients with clinical-radiological suspicion of SNPT were enrolled, and the presence of spontaneous sputum was negatively associated with SNPT [14].

In relation to radiological signs, we found in a multivariate model that having no radiographic pattern typical of TB was statistically associated with a diagnosis of SNPT. Atypical chest x-ray patterns or even normal findings were more frequent in SNPT cases as demonstrated previously by other authors [32,33]. In a study [33] that included all culture-confirmed, pulmonary TB cases reported to the US National TB Surveillance System from 1993 to 2008, SNPT cases were more likely to present atypical radiographic findings. Typical radiological patterns of pulmonary TB reactivation are less often observed in SNPT patients due to the smaller burden of mycobacteria [27], especially in areas with high prevalence of HIV infection. In these settings, clinicians are less likely to find typical radiological signs of pulmonary TB, and the diagnosis of TB is therefore more challenging [34].

Although approximately 40% of our study sample is HIV positive, no statistically significant association was observed between SNPT and HIV infection. In HIV high-prevalence countries, the increase of SNPT is thought to contribute significantly to delays in TB diagnosis and treatment [34–36]. Additionally, SNPT was originally thought to have a better prognosis compared with SPPT, however the HIV epidemic has led to changes in the presentation of SNPT disease from a slowly progressive disease to one with high mortality rate [37,38]. Previous studies demonstrated that HIV positive patients are more likely to have SNPT than HIV negative ones [6,17,23,39], and this probability increases as immunosuppression increases [34]. Nonetheless, other authors [14,31] have also found no association between HIV infection and SNPT. One of these studies [14] was also conducted in Brazil, including patients with clinical-radiological suspicion of SNPT to develop a prediction model for SNPT for outpatients. HIV infections or AIDS were not among the variables significantly associated with a diagnosis of SNPT.

One of the limitations of this study is that we recruited patients from a single hospital, and the results may thus not apply to other settings. Furthermore, the information obtained retrospectively from chart review may be less complete than data collected prospectively. Also, it would be interesting to build a predictive model and evaluate the utility of the identified clinical features. In addition, we could not demonstrate excess early mortality in SNPT vs SPPT because our sample size was not calculated to identify this difference. Probably a larger sample would be needed to demonstrate it. However, the study was not designed with this purpose. In spite of these concerns, the knowledge of the epidemiological characteristics, clinical presentation, and radiological findings of patients with SNPT is important to define the characteristics that could be used to identify individuals with confirmed SNPT.

In conclusion, we found a very high prevalence of SNPT among patients with TB in a setting with high TB and HIV prevalence. The absence of cough in the presence of other symptoms suggestive of TB, and having no radiographic pattern typical of TB where independent predictors of SNPT. Future prospective studies are warranted in this setting to create clinical scoring algorithms to help to identify and correctly to treat patients with SNPT.
